# Unraveling the signaling pathways of plant cold stress: current insights and future directions

**DOI:** 10.3389/fpls.2025.1666852

**Published:** 2025-09-24

**Authors:** Chen Peng, Wei Hua, Jing Liu

**Affiliations:** ^1^ Key Laboratory of Biology and Genetic Improvement of Oil Crops, Oil Crops Research Institute of the Chinese Academy of Agricultural Sciences, Ministry of Agriculture and Rural Affairs, Wuhan, China; ^2^ Hubei Hongshan Laboratory, Oil Crops Research Institute of the Chinese Academy of Agricultural Sciences, Wuhan, China

**Keywords:** cold stress, signaling pathway, regulatory mechanism, intelligent breeding, cold responsive gene

## Abstract

Cold stress is a major abiotic stress that seriously hinders plant growth and development, ultimately affecting crop yields. During the process of evolution, plants have evolved sophisticated adaptive strategies encompassing acclimation processes and tolerance mechanisms. Over the past two decades, substantial research breakthroughs have been made in elucidating the core components and complex regulatory networks underlying cold tolerance. This review systematically synthesizes the recent progress in three fundamental aspects: cold stress perception and signal transduction pathways, downstream physiological and molecular responses, and the pivotal regulatory roles of transcription factors (particularly CBF/DREB1 family) and cold-responsive miRNAs. In addition, we also investigated the intricate crosstalk between cold response and other biological processes including photoperiod sensing, flowering regulation, circadian rhythm, phytohormone signaling, and the dedicated discussion addresses how plants achieve metabolic and developmental trade-offs when allocating resources between cold defense and other vital traits. Looking forward, we propose four promising research directions: identifying novel cryo-sensors beyond currently known receptors, post-translational modification dynamics of CBF proteins, homeostatic control mechanisms among competing regulatory factors, and translational applications of cold stress pathways in precision breeding programs. Addressing these knowledge gaps will not only deepen our understanding of plant cold adaptation at molecular level, but also facilitate the development of climate-resilient crops through molecular design breeding.

## 1 Introduction

Cold stress is a significant factor limiting plant growth and development, which often leads to a slowdown or even arrest of plant growth, and ultimately results in a large-scale decline in grain crop yields (Shi et al., 2018). For example, cold stress can cause a decrease in tomato yield by 8–21%, rice yield by 15–35%, and chickpea, soybean, and mung bean yields by 45–61% respectively (El-Refaee et al., 2024; Oliveira et al., 2025). Based on the different physiological mechanisms that function at varying temperature levels, the stress can be divided into chilling stress (0-15 °C) and freezing stress (below 0 °C) (Chinnusamy et al., 2007). Chilling stress mainly results from osmotic dehydration caused by extracellular ice crystals, leading to the hardening of plant membranes, the destruction of organelles and the inhibition of plant growth (Wang et al., 2024c). However, plants suffer greater damage under freezing stress because water diffuses out of cells and forms ice crystals, decreasing extracellular water potential and causing severe dehydration and ultimately death (Villouta et al., 2021).

Plants have evolved adaptive strategies to combat cold stress through prolonged evolutionary processes. Tropical plants such as rice can enhance the cold tolerance after a period of cold treatment. Temperate plants such as rapeseed, wheat, and *Arabidopsis* usually exhibit a higher tolerance to cold stress than tropical plants. The phenomenon of improving cold tolerance through cold training is called cold acclimation (Kutsuno et al., 2023). During this period, many changes occur, including upregulation and downregulation of related genes, which will alter the levels of some proteins (such as AFPs), metabolites, and hormones in order to combat cold stress (Maruyama et al., 2012). In recent years, research on cold tolerance has attracted widespread attention, and many cold related genes have been discovered. However, there are few reports that comprehensively explain the molecular mechanisms of how plants respond to cold stress from multiple dimensions. Therefore, this article will focuses on the cutting-edge dynamics of plant cold tolerance mechanisms, including perception, signal transmission, and response. It emphasizes the analysis of key gene functions closely related to cold stress and provides a systematic classification and detailed explanation of related signaling pathways. It has irreplaceable value in deepening our understanding of how plants can cleverly avoid or reduce cold damage. Meanwhile, the article also sorted out problems that need to be urgently solved and, in combination with the existing cold stress related gene information, put forward practical and feasible technical suggestions for the practice of cold-resistant molecular breeding, aiming to provide useful references for scientific research and application in this field.

## 2 Cold signal perception

The cell membrane serves as the primary site for plants to perceive temperature, with cold signal perception commencing via multiple receptors located on the membrane. To date, several potential cold sensors/receptors have been identified. In *Arabidopsis*, the plasma membrane receptor-like kinase CRLK1 modulates Ca^2+^ channel activity, thereby triggering calcium signal transduction and activating the MAPK kinase cascade reaction during the period of cold stress. This ultimately leads to the upregulation of *CBF1* expression, thereby enhancing plant cold tolerance (Zhao et al., 2017). Additionally, the cytoplasmic receptor-like kinase CRPK1, located on the plasma membrane (PM), facilitates its interaction with CBF proteins by specifically phosphorylating 14-3–3 proteins, and actively regulates cold tolerance (Liu et al., 2017). Recent studies have indicated that the formation of a complex between CRPK1 and the receptor protease KOIN influences the response of the 14-3-3-CBF module to cold stress (Zhang et al., 2025). These cold receptors connect cold perception with the subsequent cold transduction.

Cold sensors are equally vital for cold perception across different plant species. For instance, in rice, the G protein regulatory factor COLD1, which is located in the plasma membrane, forms a functional complex with RGA1. This complex not only mediates the initial perception of cold signals but also induces extracellular Ca^2+^ influx, thereby activating downstream signaling cascades (Ma et al., 2015b). Recent research indicates that OsSRO1c forms biomolecular aggregates through liquid-liquid phase separation (LLPS), recruiting the transcription factor OsDREB2B into nuclear aggregates. This dynamic phase transition significantly enhances cold tolerance in rice by promoting the expression of COLD1 (Hu et al., 2024b). In *Vitis amurensis*, VaCOLD1 also actively regulates cold stress. VaCOLD1 forms a complex with the G protein α subunit VaGPA1, activates Ca^2+^ channels, promotes an increase in intracellular Ca^2+^ concentration, and triggers the MAPK cascade reaction and the downstream CBF signaling pathway (Zheng et al., 2023). Collectively, COLD1 plays a conserved yet flexible role as a cold sensor in both monocotyledonous and dicotyledonous plants, which providing a crucial molecular target for cold tolerance crop breeding.

The Ca^2+^ channels can also function as cold receptors by sensing cold stress. These channels regulate the transmembrane flow of Ca^2+^ in cellular perception of cold signals. In *Arabidopsis*, the permeable transporter protein ANN1, located on the plasma membrane, mediates cold-induced Ca^2+^ influx into the cytoplasm, thereby establishing frost resistance (Liu et al., 2021a). The calcium-permeable channel AtMCA1/2 is involved in increasing cold-induced Ca^2+^ concentration and enhancing cold tolerance (Mori et al., 2018). The Ca^2+^-permeable channel CNGC20 actively regulates frost resistance in *Arabidopsis* by influencing cold-induced Ca^2+^ influx. Deletion of the CNGC20 gene results in reduced Ca^2+^ influx. Similarly, the rice homolog OsCNGC20 positively regulates plant cold tolerance by affecting Ca^2+^ flow. During initial cold exposure, receptor kinase PSY1R phosphorylates OsCNGC20, inducing cytosolic Ca^2+^ influx. In later stress phases, CRPK1 phosphorylates CNGC20 and triggers its degradation via the 26S proteasome. Thus, PSY1R and CRPK1 regulate OsCNGC20-mediated cold stress in an antagonistic manner (Peng et al., 2024a). Another channel, OsCNGC9, a homolog of the *Arabidopsis* OST1 gene, can be activated by phosphorylation, triggering cytoplasmic calcium influx and activating the downstream *cold responsive genes* (*CORs*) expression (Wang et al., 2021). The mechanism by which plants perceive cold stress through plasma membrane ion channels exhibits both significant conservation and diversity.

## 3 Cold signal transduction

**Figure 1:**
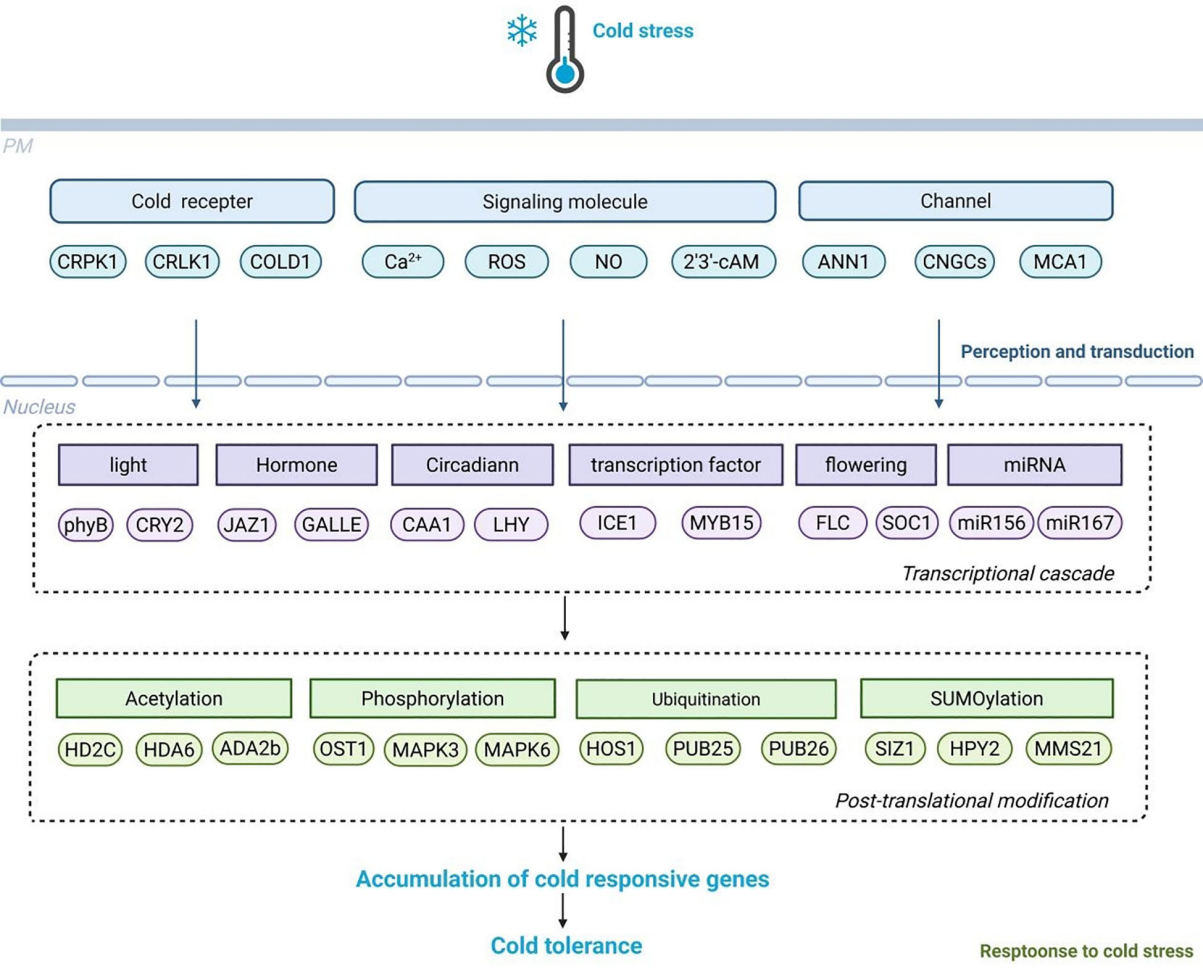
The signal perception, transduction, and response mechanisms of plant cold stress. Plants perceive cold signals through cold receptors such as COLD1, and conduct signal transduction through signaling molecules such as Ca2+, NO, ROS, as well as related channels such as ANN1 and MCA1. These signaling molecules activate a series of transcription factors, circadian rhythms, light, flowering, and enzyme related regulatory factors in the transcription cascade and post-translational modifications to induce the expression of COR genes under cold stress.

After being perceived, cold signals undergo a series of signal molecules and multiple transmission pathways to achieve cold signal transduction (Mortazavi et al., 2008). Secondary messengers like Ca^2+^, ROS, and NO are generated by cells to convey cold signals. These messengers control intracellular Ca^2+^ levels, resulting in Ca^2+^ influx and subsequent signal transduction (**Figure 1**).

### 3.1 Ca^2+^ signal

Ca^2+^ serves as the second messenger in plant cells, mediating various developmental processes and responding to environmental stimuli by triggering primary signals (Yamazaki et al., 2008). Under cold stress, cell membrane channels are activated, causing Ca^2+^ influx. These Ca^2+^ signals are recognized by Ca^2+^-dependent protein kinases such as CIPKs, CaM, CAMTAs, and CDPKs, which interpret the signals and initiate subsequent events. In *Arabidopsis*, the Ca^2+^-dependent protein kinase CIPK3 is activated after cold stimulation, directly phosphorylating CNGC5/6 channels, enhancing their permeability, and promoting initial Ca^2+^ influx. CaM2 binds to CNGC5/6 after Ca^2+^ accumulation, inhibiting channel activity and preventing cell toxicity caused by Ca^2+^ overload (Ming et al., 2025). CPK28, another Ca^2+^-dependent protein kinase, can be rapidly activated and regulates the expression level of *CBF* genes via phosphorylating nin-like protein 7 (NLP7) (Ding et al., 2022). Additionally, Ca^2+^-dependent protein kinase CAMTA3/5 can induce the expression of the *CBF1* gene, helping plants combat rapid temperature drops (Kidokoro et al., 2017). Based on the above research, it can be clearly seen that Ca^2+^ acts as a key mediator of cold signaling, and Ca^2+^-dependent protein kinases serve as a vital link between Ca^2+^ signal transduction and *CBF* gene expression.

The relationship between Ca^2+^ and cold stress in other plants has also garnered attention. In tomato, low temperatures can trigger Ca^2+^ influx. The Ca^2+^ signal is captured and decoded by SICaM6 before entering the nucleus, binds to ICE1 and inhibits its transcriptional activity, ultimately reducing tomato cold tolerance (Lin et al., 2023). In rice, OsCPK24 negatively regulates the cold tolerance via phosphorylating OsGrx10, maintaining high levels of glutathione and phosphorylation, and thereby enhancing cold tolerance (Liu et al., 2018). Despite significant progress in studying Ca^2+^ signaling mechanisms under cold stress, the principles and dynamic changes of Ca^2+^ production during cold stress process remain unclear. Exploring the spatiotemporal dynamics of Ca^2+^ is vital for elucidating the specific mechanisms of Ca^2+^ signal transduction in plant adaptation to cold stress.

### 3.2 Reactive oxygen species signal

Cold stress can cause damage to the cell membrane system, increase membrane permeability, lead to electron leakage, and induce the production of ROS, including hydrogen peroxide (H_2_O_2_), superoxide anion (O_2-_), hydroxyl radicals (OH-) (Mittler, 2002). The role of ROS depends on its concentration level. When plants are under optimal growth conditions, intracellular ROS levels are low. However, under cold stress, excessive accumulation of ROS can cause oxidative damage by attacking polyunsaturated fatty acids, leading to lipid peroxidation and altering membrane fluidity. ROS can also oxidase and modify amino acid residues in proteins, altering protein activity, thereby affecting the function of transcription factors, regulating downstream gene expression and ultimately causing serious impact on plant growth (Gill and Tuteja, 2010).

Maintaining a low ROS concentrations in plants is widely recognized as beneficial for growth. In rice, the mitochondrial protein SOP10 can regulate superoxide generation and positively regulate cold tolerance. Research has shown that the *sop10* mutant exhibits significantly enhanced survival ability at low temperatures due to reduced ROS accumulation (Zu et al., 2023). In apple, MdNAC104 improves cold tolerance by regulating ROS levels and enhancing antioxidant capacity. Transgenic plants overexpressing MdNAC104 show decreased ion leakage and ROS accumulation under cold stress, while osmotic regulatory substances and antioxidant enzyme activity increase (Mei et al., 2023). These findings highlight the important role of ROS in plant cold stress responses, providing potential gene targets for developing cold-resistant plant varieties and promoting research in plant cold tolerance breeding.

### 3.3 Nitric oxide signal

NO also acts as a messenger molecule and is widely involved in cold response (Wilson et al., 2008). At low levels, NO acts as a signaling molecule, while at high levels, it induces cell damage and triggers NO stress. In *Arabidopsis*, cold acclimation promotes the production of endogenous NO and the accumulation of osmotic substance. In the *nia1nia2* double mutant, the expression levels of *CBF1/2/3*, *KIN1*, and *COR15a* were significantly decreased, indicating that NO is a necessary signal for rapid upregulation of cold-induced genes (Cantrel et al., 2011). Further research reveals that NO actively regulates 14-3–3 protein expression and mediates the CBF/DREB1 transcription network (Yang et al., 2013b).

Previous studies have shown that NO-induced protein S-nitrosylation prevent cold damage by regulating plant antioxidant mechanisms. NO mediates the S-nitrosylation of superoxide dismutase (SOD), enabling it to regulate ROS detoxification through the antioxidant system (Zhao et al., 2009). When cold stimulation triggers the opening of the plasma membrane Ca^2+^ channel MCA1/2, NO activates calcium dependent protein kinase (CPK) through S-nitrosylation modification to enhance calcium transients and promote transcriptional bursts in the CBF regulatory network In wheat (Babuta et al., 2025). The precise interaction between NO and ROS also constitutes the defense line against cold in plants. Cold stress can trigger explosive accumulation of ROS, and NO acts not only as a scavenger for ROS but also maintain redox balance by activating antioxidant enzymes such as SOD. In dichondra, cold stress leads to a rapid increase of NO in cold resistant varieties, causing soluble sugar accumulation. The antioxidant system works together to achieve low ROS accumulation, while cold sensitive genotype varieties lack this linkage mechanism (Ling et al., 2025).

The relationship between NO and Ca^2+^ under cold conditions has attracted attention. At low temperatures, the concentration of Ca^2+^ rapidly increases in the cytoplasm, leading to an enhanced expression of nitrate reductase ClNR1 and a sharp increase in NO. In turn, NO induces the expression of the cyclic nucleotide-gated channel ClCNGC20, which further causes Ca^2+^ influx. This ultimately inhibits the negative regulatory module of ClCaM2/5/7-ClVDAC1, thereby maintaining high CBF pathway activity and significantly enhancing cold resistance in watermelon, indicating that Ca^2+^ and NO have a synergistic effect in plant cold response (Guo et al., 2025).

### 3.4 2’,3’-cAMP signal

2’,3’-cAMP is emerging as a novel second messenger. Earlier studies have revealed that 2’,3’-cAMP synthase activity resides in TIR domain proteins, which are pivotal in plant immune responses (Yu et al., 2022). Recent advances have highlighted the critical role of 2’,3’-cAMP in cold reaction. Under cold stress conditions, COLD6 forms a protein complex with cold-induced OSM1. This complex senses extracellular cold signals, triggers the accumulation of 2’,3’-cAMP and subsequently activates the *COR* genes expression (Luo et al., 2024). Notably, studies have observed a high degree of synchronization between the accumulation of 2’,3’-cAMP and the expression of *COR* genes in *Arabidopsis* over time. This finding further reinforces the importance of 2’,3’-cAMP in cold signal transduction. Moreover, the application of exogenous 2’,3’-cAMP analogs can mimic the effect of increased intracellular 2’,3’-cAMP levels and enhance cold tolerance. This suggests that 2’,3’-cAMP can function as a signaling molecule to trigger cellular cold defense responses. In addition, 2’,3’-cAMP may amplify cold signals and activate downstream *COR* gene expression by interacting with CaMs and CDPKs in the calcium signaling pathway. Future exploration of the relationship between 2’,3’-cAMP and other messenger molecules promises to be highly insightful.

## 4 Cold signal response

cold signals are transmitted from the PM to the nucleus, triggering transcription and post transcriptional regulation, inducing the expression of cold related genes, and ultimately affecting plant cold tolerance. In the past two decades, various regulatory pathways have been discovered in plants at different stages to respond to cold stress, and key genes in these pathways have been continuously revealed (**Table 1**). These regulatory pathways are crucial for plants to combat cold stress. Next, we will describe the regulation of transcription factor, flowering, circadian rhythm, hormone regulation, and miRNA in the transcriptional cascade, as well as the regulation of phosphorylation, ubiquitination, and acetylation in post-translational modifications. These regulatory factors collectively respond to cold stress.

**Table 1:** Representative key genes in different pathways related to cold stress.

Pathway	Gene	Target	Effect on freezing tolerance	Transgenic plant	References
Ca^2+^	*CAMTA3*	CBF2	positive	*Arabidopsis*	(Doherty et al., 2009)
	*CaM4*	COR	negative	*Arabidopsis*	(Chu et al., 2018)
	*AtANN1*	CBFs	positive	*Arabidopsis*	(Liu et al., 2021a)
	*OsCNGC20*	CBFs	positive	Rice	(Peng et al., 2024b)
	*OsCPK24*	OsGrx10	positive	Rice	(Liu et al., 2018)
	*CaM6*	ICE1	negative	Tomato	(Lin et al., 2023)
Circadian	*CCA1/LHY*	CBFs	positive	*Arabidopsis*	(Dong et al., 2011)
	*TOC1*	LKP2	positive	*Arabidopsis*	(Maeda et al., 2024)
	*RVE4*	DREB1s	positive	*Arabidopsis*	(Kidokoro et al., 2023)
	*PRR7*	CBFs	negative	*Arabidopsis*	(Nakamichi et al., 2009)
Flowering	*VIN3*	FLC	positive	*Arabidopsis*	(Zhao et al., 2020)
	*FRIGIDA*	FLC	positive	*Arabidopsis*	(Zhu et al., 2021)
	*SOC1*	CBFs	negative	*Arabidopsis*	(Seo et al., 2009)
Phytochroe	*phyA/B*	PIF4/7	negative	*Arabidopsis*	(Wang et al., 2016)
Hormone	*JAZ1/4*	ICE1	negative	*Arabidopsis*	(Hu et al., 2013)
	*NPR1*	HSFA1	positive	*Arabidopsis*	(Olate et al., 2018)
	*AHK2*	AHPs	negative	*Arabidopsis*	(Jeon and Kim, 2013)
	*CRF2*	PINs	positive	*Arabidopsis*	(Jeon et al., 2016)
	*BZR1*	CBFs	positive	Tomato	(Fang et al., 2021)
Transcription factor	*ICE1*	CBF3	positive	*Arabidopsis*	(Chinnusamy et al., 2003)
	*CBF1/3*	CORs	positive	*Arabidopsis*	(Novillo et al., 2007)
	*PIF3*	CBFs	negative	*Arabidopsis*	(Jiang et al., 2017)
	*CES*	CBFs/COR	positive	*Arabidopsis*	(Eremina et al., 2016)
	*MYB15*	CBFs	negative	*Arabidopsis*	(Agarwal et al., 2006)
	*WRKY41*	CBFs	negative	*Arabidopsis*	(Wang et al., 2023b)
	*MdNAC104*	CBF1	positive	Apple	(Mei et al., 2023)
	*MdMYB124*	MdCCA1	positive	Apple	(Xie et al., 2018)
	*CtrTGA2*	CtrP5CS1	positive	Citrus	(Xiao et al., 2024)
	*MtNAC80*	MtGSTU1	positive	Medicago truncatula	(Ye et al., 2024)
	*OsNAC5*	OsABI5	positive	Rice	(Li et al., 2024b)
	*OsSRO1c*	DREB2B	positive	Rice	(Hu et al., 2024b)
	*bZIP73*	bZIP71	positive	Rice	(Liu et al., 2019)
	*SIHY5*	SlGA2ox4	positive	Tomato	(Wang et al., 2019a)
	*ERF15*	CBF1	positive	Tomato	(Hu et al., 2024a)
	*SlBBX17*	CORs	positive	Tomato	(Song et al., 2023)
Enzyme	*MPK3/6*	ICE1	negative	*Arabidopsis*	(Zhao et al., 2017)
	*BIN2*	ICE1	negative	*Arabidopsis*	(Ye et al., 2019)
	*OST1*	ICE1	positive	*Arabidopsis*	(Ding et al., 2015)
	*PUB25/26*	MYB15	positive	*Arabidopsis*	(Wang et al., 2019b)
	*HOS1*	ICE1	negative	*Arabidopsis*	(Dong et al., 2006)
	*SIZ1*	HOS1	positive	*Arabidopsis*	(Miura et al., 2007)
	*OsSAPK6*	IPA1	positive	Rice	(Jia et al., 2022)

### 4.1 Transcription factor responds to cold stress

Transcription factors are key regulatory proteins situated at the forefront of plant growth regulation networks. Numerous transcription factor families in plants, such as AP2/ERF, WRKY, NAC, MYB, and bZIP, are known to be activated in response to cold stress.

The CBF/DREB transcription factor is a member of the AP2/ERF superfamily and serves as a core regulatory hub for cold response. In *Arabidopsis*, CBF proteins bind to CRT/DRE cis-elements within the promoters of *COR* genes (including *COR*, *LTI*, and *RD*) to activate transcriptional programs that enhance freezing tolerance. Genetic evidence indicates that *cbf1/cbf2/cbf3* triple mutants exhibit hypersensitivity to freezing stress, accompanied by compromised seed germination and impaired stress-responsive capacity. Notably, AtCBF4 has been identified primarily as a mediator of drought stress adaptation (Shi et al., 2018). Recent advancements have revealed that CBF protein accumulates under cold conditions and directly interacts with SKIP, a key component of spliceosomes. SKIP serves as an essential factor regulating alternative splicing. CBF facilitates the formation of nuclear liquid-liquid phase separation aggregates involving SKIP proteins through its intrinsic disordered domain (IDR). This process enriches transcripts from the COR gene family and significantly enhances their alternative splicing efficiency. However, disrupting the interactions between CBF and SKIP or inhibiting phase separation markedly diminishes plant frost resistance. These findings underscore that regulation of alternative splicing induced by cold stress is critical for effective cold response (Fu et al., 2025).

In other plants, CBFs also showed a positive effect in response to cold stress. In rice, OsCBF3 positively regulates cold tolerance (Wang et al., 2021). The expression of OsCBF1/2/3 is directly regulated by OsERF52. Further research has found that OsSAPK9 can directly phosphorylate the Ser261 site of OsERF52 to promote protein accumulation, thereby increasing the transcription level of OsCBF1/2/3 (Xu et al., 2024a). In sweet potato, Overexpression of *CBF3* gene enhances the cold tolerance. This indicates that CBF3 positively regulates the cold tolerance (Jin et al., 2017). In summary, CBF transcription factors exhibit conservation and species diversity.

The CBF-dependent pathways, especially the ICE1-CBF-COR pathway, is a key regulatory pathway conserved across different plant species (Tang et al., 2020). Under cold stress, ICE1 is activated and induces *CBF* expression by binding to the *CBF3* promoter. The CBF protein further activates downstream *COR* genes, thereby enhancing cold tolerance. ICE1 stability is regulated by ubiquitination via HOS1 and SUMOylation by SIZ1 (Dong et al., 2006; Miura et al., 2007). Homologs of ICE1, such as OsbHLH002 in rice, also enhance plant cold tolerance by regulating the *OsCBFs* expression (Zhang et al., 2017; Xu et al., 2024a). Recent studies have revealed that the transcription factor AIF2 acts as a key mediator of the CBF-dependent pathway. Specifically, AIF2 enhances the stability of ICE1, thereby promoting the transcription of *CBF* genes, which collectively increase the cold tolerance. Additionally, AIF2 physically interacts with and induces phosphorylation-dependent degradation of MPK3/6 (Kim et al., 2024), which were previously shown to negatively regulate cold tolerance by phosphorylating ICE1 and targeting it for proteasomal degradation (Li et al., 2017a).

Despite the CBF-dependent pathway is important, it only regulates approximately 12% of *COR* genes. Many *COR* genes exhibit CBF-independent expression under cold stress, suggesting that plants may employ a multi-level transcriptional regulatory network involving other transcription factor families, such as bZIP, MYB, NAC, and WRKY.

The bZIP transcription factor is highly conserved across multiple species and is widely involved in plant stress response and adverse conditions. The conserved sequence of bZIP proteins (PYCGTGG) can specifically bind to the ABRE element (ACGT) in the promoters of target genes. In *Arabidopsis*, the bZIP transcription factors elongated hypocotyl 5 (HY5) and its homolog HYH activate gene expression by binding to the G-box element (ACGTGTC) in the *SIG5* promoter. This process regulates the transcription of the chloroplast gene psbD BLRP, maintaining the abundance of the PSII D2 protein and thereby sustaining photosynthetic efficiency under long-term low-temperature and short-term freezing conditions (Cano-Ramirez et al., 2023). Similarly, in maize, HY5 binds to the natural variation site of the *COOL1* gene promoter to regulate *COOL1* expression, which affects adaptability to high-latitude and cold stress. The CPK17 kinase, activated by cold stress, stabilizes the COOL1 protein through phosphorylation, thereby enhancing cold tolerance (Zeng et al., 2025). In asparagus beans, VunHY5 and VunMED2 synergistically bind to the G-box/BRE element in the *VunERD14* promoter, inducing its expression and increasing the expression of antioxidant genes to actively regulate cold tolerance (Liang et al., 2025). Likewise, overexpression of *SlHY5* in tomatoes can elevate the expression levels of antioxidant enzyme genes, reduce ROS content, and enhance plant cold tolerance. Notably, SlHY5 can also influence tomato cold tolerance by directly regulating the expression of *COR* genes. It is important to highlight that bZIP family members exhibit bidirectional regulatory characteristics in cold response. Recent studies have shown that SlHY5 can directly affect CBF transcription levels and indirectly regulate *CBF* expression by inhibiting MYB15 (Zhang et al., 2020). Additionally, the cold-activated MAPK family member SlMPK1/2 phosphorylates the SlBBX17 protein, enhancing its interaction with SlHY5 and thereby synergistically improving cold tolerance (Song et al., 2023). In contrast, in maize, bZIP68 acts as a negative regulatory factor. After phosphorylation modification of ZmMPK8, cold tolerance is reduced by inhibiting the expression of *ZmDREB1* gene (Li et al., 2022). However, the natural variation of the *HSF21* gene promoter can alleviate the transcriptional repression of bZIP68 and compensate for cold tolerance by increasing the expression level of *HSF21* induced by cold stress. The research on rice further expands the regulatory dimension of bZIP. The heterodimer formed by bZIP73Jap and bZIP71 enhances cold tolerance during seedling stage by activating peroxidase gene expression, while during reproductive growth stage, it specifically enhances the expression of sugar transport related genes such as *OsMST7*, *OsMST8*, and *OsINV4*, significantly improving the seed setting rate under cold stress by promoting soluble sugar transport from anthers to pollen (Liu et al., 2019).

MYB transcription factors constitute one of plant biology’s most expansive TF families, functioning as master regulators coordinating developmental programs and abiotic stress adaptation pathways. In *Arabidopsis*, AtMYB15 acts as a crucial regulator in response to cold stress. Mutant plants lacking MYB15 exhibit enhanced tolerance to cold stress, whereas plants overexpressing MYB15 show reduced freezing tolerance. This is attributed to the ability of MYB15 to directly bind to the *CBF* promoter, thereby inhibiting the expression of *CBF* genes. Additionally, MYB15 can interact with the CBF regulatory factor ICE1 to modulate *CBF* genes expression (Wang et al., 2023c). However, studies on homologous mechanisms in tomatoes have revealed contrasting results. SlMYB15 in tomatoes directly binds to the *CBF* promoter and positively regulates the cold tolerance (Zhang et al., 2020). In rice, OsMYBS3 negatively regulates cold tolerance by inhibiting the expression of *OsDREB1B* (Su et al., 2010). In apple, MdMYB23 displays unique functions. It can directly bind to the promoters of *MdCBF1/2*, activating their expression. Moreover, it interacts with the promoter of *MdANR*, a key regulator of anthocyanin biosynthesis, to enhance *MdANR* expression, which in turn promotes anthocyanin accumulation and scavenging of ROS (An et al., 2018). Furthermore, MdMYB88/124 in apple activates *CBF3* expression by directly binding to the promoter of *MdCCA1*. This ultimately induces the expression of *COR* genes, thereby enhancing cell membrane stability and osmotic regulation capabilities. Additionally, MdMYB88/124 can directly bind to the promoters of key anthocyanin synthesis genes, such as *MdUFGT*, promoting anthocyanin accumulation under cold conditions through a CBF-independent pathway (Liu et al., 2021b).

NAC transcription factors comprise the dominant plant-specific regulator family. NAC proteins regulate the expression of target genes by binding to the core sequence (CGTG/A) (Shao et al., 2015). In *Arabidopsis*, NAC056 positively modulates the expression of *CBF* genes, thereby enhancing plant cold tolerance (Xu et al., 2024b). In apple, MdNAC104 directly binds to the promoters of *MdCBF1/3* to enhance the cold tolerance. Additionally, it stimulates anthocyanin accumulation under cold conditions by upregulating the expression of genes involved in anthocyanin synthesis (Mei et al., 2023). In rice, OsNAC5 actively regulates germination and seedling cold tolerance by directly activating the expression of *OsABI5*. The expression of *COR* genes is significantly upregulated in OsNAC5-overexpressing lines, while it is downregulated in *abi5* knockout mutants (Li et al., 2024b). In short, NAC transcription factors in plants exhibit distinct but effective strategies to enhance plant cold tolerance.

The WRKY transcription factor family is a highly conserved group in plants. These factors specifically recognize W-box (TTGACC/T) elements in the promoter regions of target genes. In *Arabidopsis*, AtWRKY41 directly binds to the *CBF* promoter, negatively regulating the transcription and thereby reducing freezing tolerance (Wang et al., 2023b). In contrast, the homologous protein ScWRKY41 in potato functions as a positive regulator of cold stress. It recruits the histone acetyltransferase ScHAC1 to the promoter of the key flavonoid metabolism gene *ScF3’H*, significantly increasing the histone H3K9ac modification level and activating *ScF3’H* expression to enhance cold tolerance (Bao et al., 2025). Similarly, rice OsWRKY76 positively regulates cold tolerance by directly activating OsDREB1B. Meanwhile, its upstream inhibitory factor OsWRKY63 forms a negative feedback loop to finely control the activity of this pathway, ensuring a dynamic balance in the cold response (Zhang et al., 2022b). These findings highlight the significant functional differentiation of WRKY family transcription factors across different species.

This functional differentiation may represent an evolutionary strategy developed by plants to adapt to diverse growth environments and cope with various cold stress. Further research into the molecular mechanisms underlying the functional differentiation of WRKY and other transcription factors is of great significance. It can help to unravel the complex network of plant responses to cold stress and provide a theoretical basis for developing crop varieties with enhanced cold tolerance.

### 4.2 MiRNA responds to cold stress

**Figure 2:**
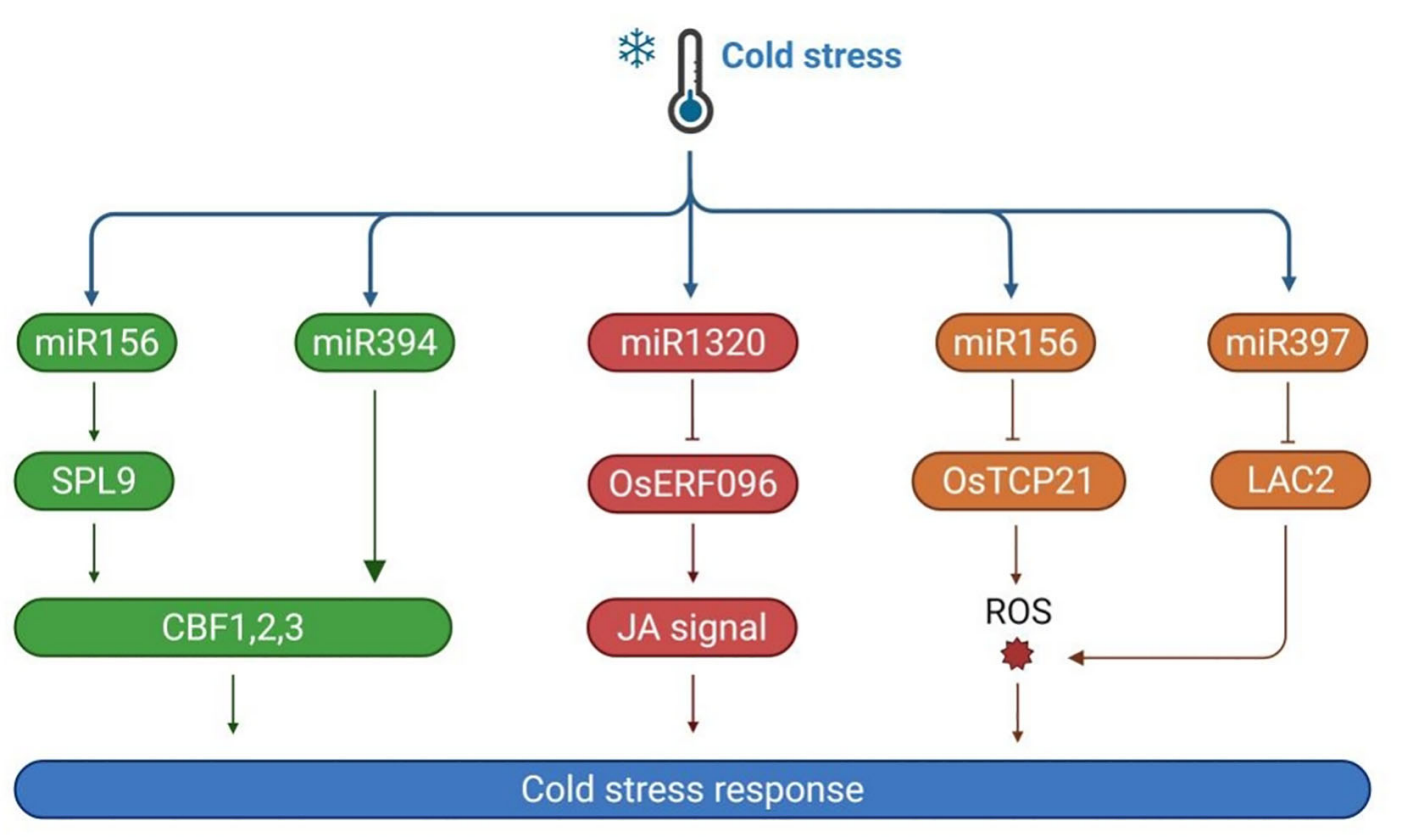
The mechanism of MiRNA responds to cold stress. When plants are subjected to cold stress, multiple miRNAs such as miR156, miR394, miR1320, and miR156 are activated. MiR156 regulates the expression of CBFs by targeting SPL9. Meanwhile, miR394 targets OsTCP21 and affects the production of ROS; MiR1320 targets OsERF096 and regulates the jasmonic acid (JA) signaling pathway. MiRNAs activate plant cold stress response mechanisms through multiple regulatory pathways, affecting plant cold tolerance.

MiRNAs represent a class of evolutionarily conserved, small non-coding RNA molecules that function as key post-transcriptional regulators in plants. A significant mode of action for miRNAs in cold response is through the CBF-dependent pathway (**Figure 2**). For instance, in *Arabidopsis*, miR319 targets the TCP transcription factor family, thereby modulating the expression of *CBF* genes and subsequently influencing the levels of COR genes (Yang et al., 2013a). Similarly, in rice, overexpression of miR319 has been shown to upregulate the expression of *CBF* genes. MiR319 can also target genes such as *OsPCF6* and *OsTCP21*, contributing to enhanced cold tolerance following cold acclimation, which suggests that miR319 may partially regulate cold tolerance in rice via the CBF-dependent pathway (Yang et al., 2013b). Other miRNAs, such as miR394, positively regulate plant cold tolerance by activating the CBF-dependent pathway. Overexpression of *CBF* genes in miR394 transgenic plants significantly enhances their expression, further supporting the role of miR394 in cold response (Huo et al., 2022). Additionally, miR397 has been demonstrated to actively regulate cold tolerance through the CBF-dependent pathway. Overexpression of miR397a in *Arabidopsis* significantly reduces electrolyte leakage in leaves, thereby enhancing cold resistance. Further research revealed an enhanced expression of *CBF* in these transgenic plants (Huo et al., 2022). MiR156 is another important miRNA involved in cold stress response. Cold stress induces miR156 expression in *Arabidopsis*, which differentially regulates its SPL targets: suppressing SPL3/13 transcripts but activating SPL9. Notably, SPL9 positively modulates *CBF2* expression to enhance the cold tolerance (Zhao et al., 2022). Similar observations have been made in bananas, where miR156e is induced under cold stress, and in sugarcane, where miR156 levels increase during cold treatment. In tomatoes, miR156 targets the cold-induced MYB15 gene, reducing cold tolerance. Overexpression of sly-MIR156e-3p increases sensitivity to cold stress, while silencing this miRNA through artificial microRNA interference enhances cold tolerance (Zhang et al., 2022a).

In addition to the CBF-dependent pathway, miRNAs also regulate plant cold tolerance by modulating ROS levels. For instance, Bna-miR397a post-transcriptionally regulates the expression of the laccase gene *BnaLAC2* in *Brassica napus*. This regulation enhances the adaptation to cold stress by reducing total lignin remodeling and maintaining ROS homeostasis (Hussain et al., 2025). The miR-397-LAC2 module has also been shown to enhance frost tolerance in *Arabidopsis*, highlighting its conserved role in cruciferous plants. MiR408 regulates cold tolerance by controlling ROS levels, with overexpression of miR408 enhancing the expression of antioxidant-related genes (Ma et al., 2015a). Furthermore, miRNAs respond to cold stress by regulating hormone signaling. In rice, miR1320 targets the ethylene-responsive factor OsERF096, normally degrading its mRNA and inhibiting its expression. Under cold stress, miR1320 expression is downregulated, leading to upregulation of *OsERF096*. This change inhibits jasmonic acid (JA) synthesis and the JA-mediated cold signaling pathway, making rice more sensitive to cold stress (Sun et al., 2022). Overall, miRNAs mediate plant cold response by integrating multiple pathways, including the CBF-dependent pathway, ROS regulation, and hormone signaling. These findings highlight the multifaceted roles of miRNAs in plant cold response and provide valuable insights for developing cold-resistant crops.

## 5 The relationship between cold stress and other factors

Beyond transcription factors and miRNAs, which are pivotal in plant cold stress responses, numerous factors including light, flowering, circadian rhythm, and hormones are also extensively implicated in plants’ intricate cold response mechanisms (Song et al., 2021).

### 5.1 Light signaling modulates cold stress response

**Figure 3:**
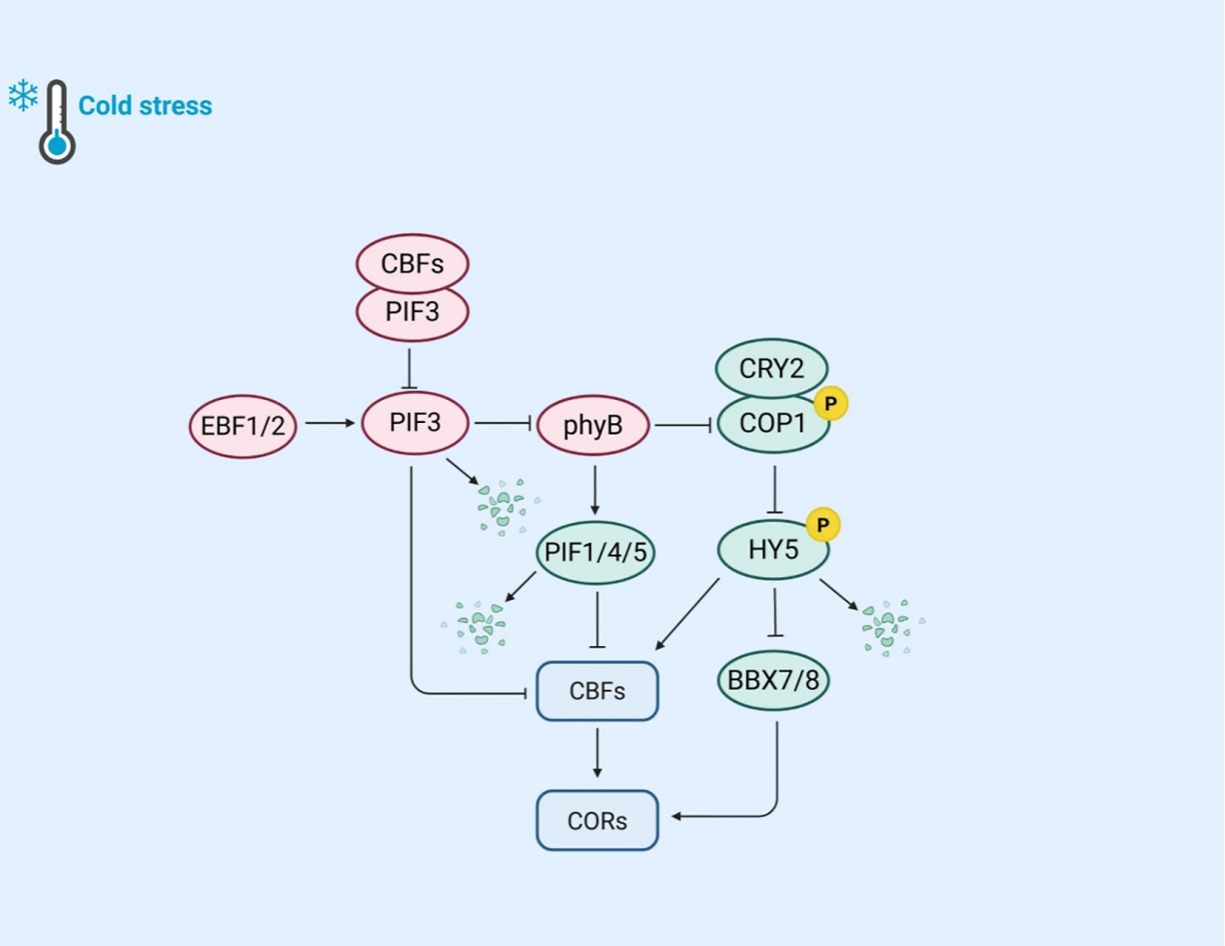
The mechanism of plant regulation of light and cold response gene expression under cold stress. Under cold stress, EBF1/2 ubiquitination degrades PIF3, reducing its inhibition of CBFs. When CBFs bind to PIF3, they can inhibit the co degradation pathway of PIF3 and phyB, thereby stabilizing the accumulation of phyB protein. Cold stable phyB further promotes the degradation of PIF1, PIF4, and PIF5, reducing the inhibition of *CBF* genes by PIFs. Meanwhile, phyB can inhibit the expression of COP1; The blue light receptor CRY2 phosphorylates COP1, effectively weakening the interaction between HY5 and COP1, thereby promoting the accumulation of HY5. HY5 responds to cold stress by directly binding to *CBF* promoters or by regulating the expression of *COR* genes through BBX7/8.

Light is a vital factor influencing plant growth and stress responses. Cold stress is a prevalent abiotic stress that substantially impacts plant survival and productivity. Plants have evolved mechanisms to integrate light and cold signals (**Figure 3**). Key regulatory factors in plant light signaling include red light photoreceptors (PHYTOCHROMES), blue light photoreceptors (CRYPTOCHROMES), and phytochrome interacting factors (PIFs).

Phytochromes, including phyA and phyB, are essential red light photoreceptors that mediate plant responses to cold stress under specific light conditions. PhyB is particularly important in regulating photoperiodism and *CBF* expression. Under long-day conditions with red or white light, *phyB* mutants exhibit significantly reduced cold tolerance, whereas plants overexpressing phyB show enhanced cold tolerance. This highlights phyB’s critical role in light and cold signal transduction. The CBFs-PIFs-phyB module is vital for integrating light and cold signals in plants. Under warm light conditions, CBFs directly interact with PIF3, preventing the synergistic degradation of PIF3 and phyB proteins. When the temperature drops, PIF3 binds to the *CBF* gene promoter, downregulating *CBF* expression and acting as a negative regulator of cold tolerance. However, CBFs can inhibit the co-degradation pathway of PIF3 and phyB, stabilizing phyB protein levels. The stabilized phyB further promotes the degradation of PIF1, PIF4, and PIF5, regulates *COR* gene expression, enhances cold tolerance, and forms a positive feedback loop to strengthen frost resistance (Jiang et al., 2020). Similar regulatory mechanisms have also been found in other plants, such as in rice, where phyB inhibits the expression of *OsPIL16* (PIF3 homologous gene), thereby suppressing the binding of OsPIL16 to the N-box region of the *OsDREB1B* promoter (He et al., 2016). Additionally, F-box proteins EBF1/2 are involved in this regulatory process. They target PIF3 for degradation via the 26S proteasome pathway. Previous research found that phyB interacts with EBF1/2, enhancing substrate E3 ligase interactions in a light-dependent manner to control EIN3 stability (Jiang et al., 2017).

CRY2 is a blue light photoreceptor whose degradation via the 26S proteasome pathway is activated by blue light under cold conditions. Studies have shown that the E3 ubiquitin ligase LRB interacts with CRY2, regulating its ubiquitination and stability, allowing plants to accurately respond to temperature changes (Ma et al., 2021). Other research indicates that CRY2 regulates plant cold tolerance through the COP1-HY5-BBX7/8 signaling module. Under cold stress, the blue light-induced phosphorylated form of CRY2 remains stable. The stable CRY2 competes with HY5 during light signal transduction, weakening the interaction between HY5 and COP1 and promoting HY5 accumulation (Li et al., 2021). BBX7/8, a B-BOX domain protein and a direct target of HY5, positively regulates frost resistance by controlling *COR* gene expression. In contrast, BBX29, another member of the BBX family, acts as a CBF-independent negative regulator of cold tolerance in *Arabidopsis* (Wang et al., 2023a). These findings suggest that CRY2 is jointly regulated by blue light and environmental temperature.

The PIF family serves as a pivotal regulator in light responses and is widely involved in cold adaptation, exhibiting diverse functions across different plant species. Under warm conditions, PIFs typically interact with phytochromes to modulate light-mediated signal transduction. However, under cold stress, PIFs adopt distinct roles. For instance, in rice, PIF family members such as OsPIL16 regulate the cold tolerance and grain shape (He et al., 2016). In maize, ZmPIF6 improves cold tolerance by reducing ROS content and enhancing cell membrane stability while increasing grain size (length and width) and thousand-grain weight (Li et al., 2024a). In tomatoes, the SlPIF4-silenced strain not only reduces fruit weight and yield, but also increases carotenoid content and accelerates ripening (Rosado et al., 2019).

### 5.2 Cold adaptation strategies in flowering regulation network

**Figure 4:**
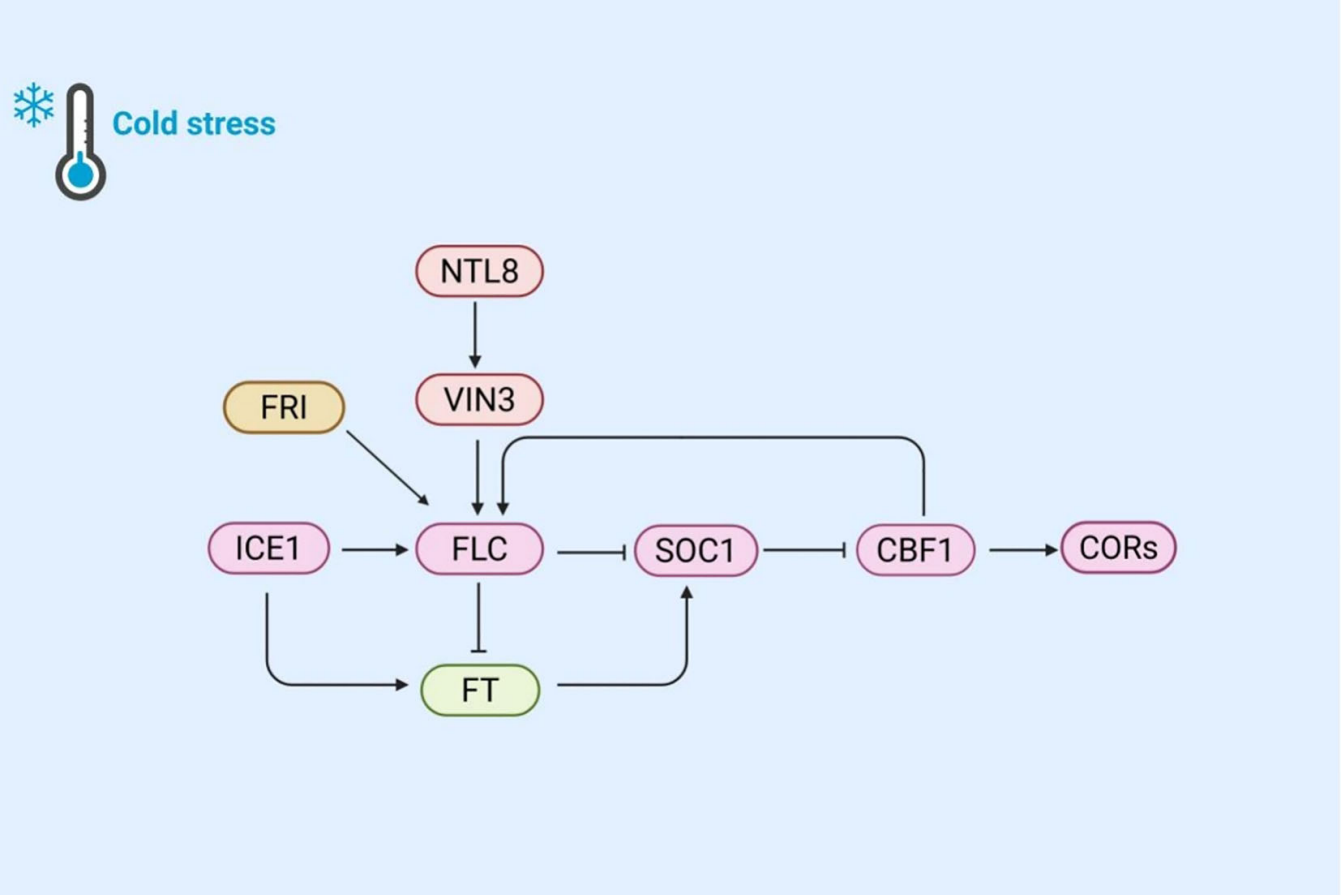
The mechanism of plant regulation of flowering time and cold response gene expression under cold stress. Cold stress activates NTL8, which in turn activates VIN3, participates in vernalization, and affects FLC expression. FRI inhibits *FLC* expression through VIN3, while ICE1 directly activates FLC. FLC inhibits SOC1 and delays flowering; SOC1 activates CBF1, promotes *CORs* expression to enhance cold tolerance. Meanwhile, ICE1 and SOC1 promote flowering through FT. This network coordinates the cold response and flowering time of plants.

More and more evidence suggests that the significance of the flowering process in plants’ response to cold stress. Winter frost, a type of cold stress, can inflict fatal damage on plant flowers and developing seeds. To mitigate this risk, most plants have evolved mechanisms to avoid flowering during winter. These mechanisms involve sensing and responding to cold signals, regulating the expression of flowering-related genes, and delaying flowering until after an extended cold period (**Figure 4**).

Under warm conditions, rapid plant growth and frequent cell division lead to the dilution of NTL8. However, under cold stress, growth retardation reduces this dilution effect, resulting in increased accumulation of NTL8 during cold exposure and the activation of *VIN3* expression (Zhao et al., 2020). The *VIN3* gene encodes a protein that inhibits the flowering inhibitory factor (FLC), thereby indirectly promoting flowering. When *FLC* expression is downregulated, plants can bloom under warm conditions. FLC acts as an upstream negative regulator of the flowering time component SOC1, which is a key flower activation factor that integrates multiple flowering induction pathways and is required for downregulating *CBF* genes. Studies have shown that in *soc1–2* knockout mutant, the expression of *CBF* genes is upregulated, whereas in *SOC1* overexpression line, their expression is downregulated (Seo et al., 2009). This indicates that SOC1 negatively regulates the expression of cold responsive genes. Cold-induced overexpression of *CBFs* can lead to late flowering by increasing *FLC* expression. Thus, a feedback loop exists between cold adaptation and flowering time regulation. When cold exposure is brief (such as in autumn or early spring), flowering is delayed due to increased *FLC* expression. However, when cold induction genes are activated by SOC1 inhibition, the cold response is suppressed. Additionally, cold-activated ICE1 directly induces *FLC* expression, which in turn inhibits *SOC1* expression and delays flowering (Lee et al., 2015). The upregulation of the *flowering locus t* (*FT*) gene in the ice1 mutant has garnered attention. FT is a key gene in the flowering pathway, and its product, the FT protein, can induce *SOC1* expression (Yoo et al., 2005). Under normal conditions, FLC inhibits FT gene expression, thereby indirectly suppressing SOC1 expression and maintaining the balance of flowering time regulation. The upregulation of the FT gene may be related to the weakened SOC1-mediated cold response. Increased FT expression enhances *SOC1* expression, which in turn negatively regulates *COR* genes. Consequently, the upregulation of the FT gene may further reduce SOC1’s inhibitory effect on cold-responsive genes, thereby affecting cold tolerance under cold stress. It is intriguing to explore whether FT is associated with the weakened cold response mediated by SOC1 and how ICE1 regulates FT gene expression.

Recent studies have shown that the *frigida* (*FRI*) gene regulates *FLC* expression at low temperatures. Cold stress can rapidly promote the formation of FRI nuclear condensates, which is associated with decreased FRI occupancy in the *FLC* promoter region and subsequent FLC inhibition (Zhu et al., 2021). This discovery reveals a potential new pathway under cold stress: the FRI-FLC-SOC1 pathway, which affects plant cold resistance. Through this pathway, plants can better balance flowering time and cold response under cold stress to adapt to environmental changes.

### 5.3 Circadian rhythm orchestrates cold stress response

**Figure 5:**
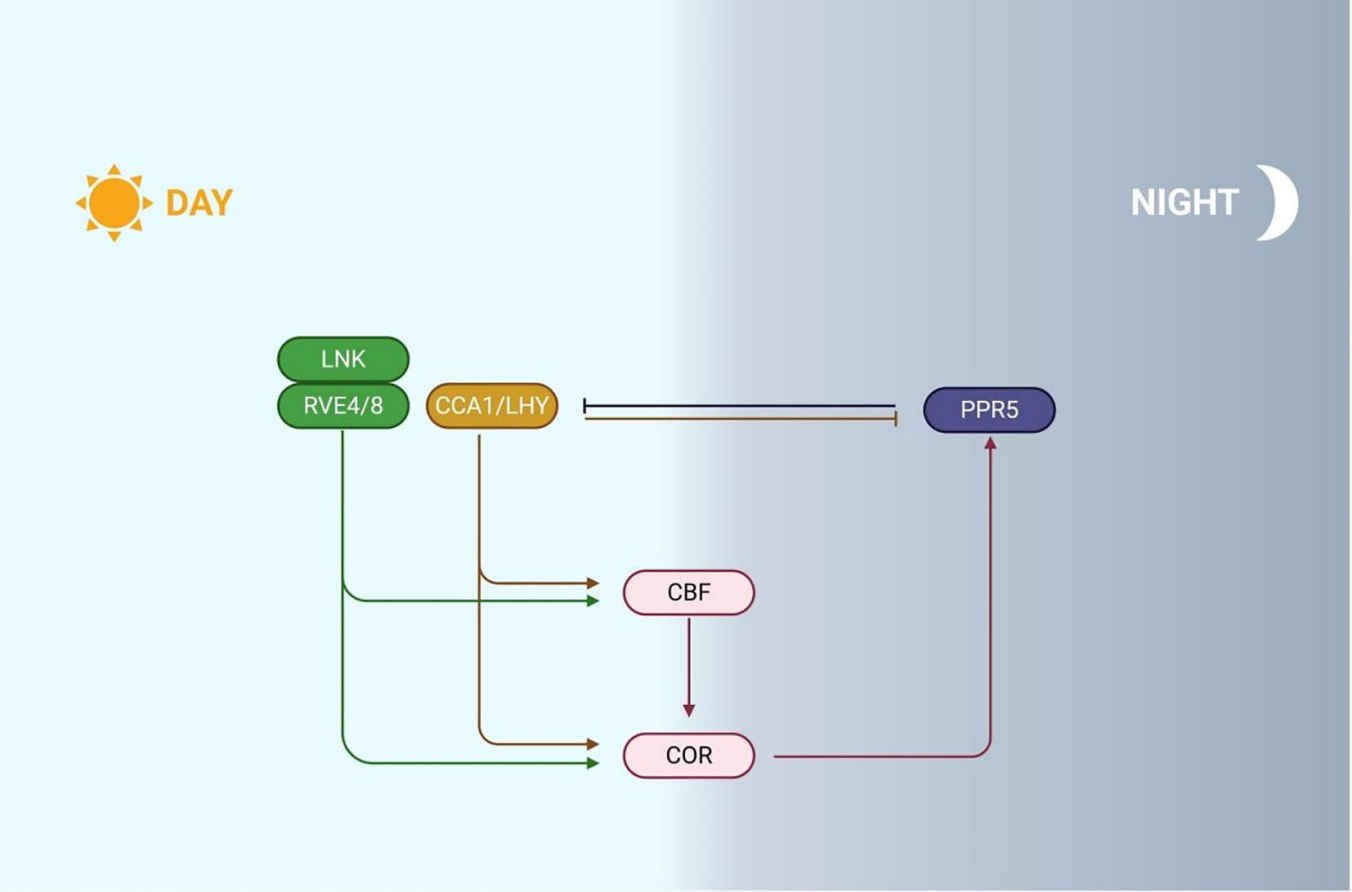
Mechanism of plant responds to cold stress through circadian rhythm. During the day, LNK works synergistically with RVE4/8 to activate CBF expression. In addition, high expression of CCA1/LHY inhibited the expression of PRRs, and as the day progressed, PRRs gradually accumulated and began to inhibit the expression of CCA1/LHY at night. At the moment, CCA1/LHY directly binds to *CBF* promoters, promoting *COR* genes expression and enhancing plant cold tolerance.

The plant circadian rhythm system is composed of key genes, including circadian clock associated 1 (CCA1), late elongated hypocotyl (LHY), lux arrhythmo (LUX), and pseudo response regulator factors (PRRs). These genes act as a positive regulator of cold response (**Figure 5**). The promoter regions of genes induced by cold stress often contain EE (evening element) and EEL elements. EE/EEL serves as the binding site for CCA1/LHY, a core component of the circadian rhythm, and its highly similar RVE protein in the target gene promoter region. CCA1 and LHY are inhibited by PRRs in the morning and bind to *CBF* promoters, actively regulating cold tolerance. For example, the tea tree homolog of LHY, CsLHY, also positively affects cold stress. Research has shown that under cold stress, the expression level of *CBFs* increases in the *prr9/7/5* mutant, and PRR5 inhibits the expression of CCA1 and LHY on cold nights (Nakamichi et al., 2009).

Under cold conditions, nuclear accumulation of CCA1 homologs RVE4/8 activates *CBF3* expression through EE element binding in its promoter (Kidokoro et al., 2021). RVE8-LNK1/2 interaction coordinately regulates circadian oscillators and *COR* genes (Sorkin et al., 2023). Independent of CBF signaling, CCA1 directly targets *COR27/28* promoters, suppressing cold-induced *COR27* expression while these genes maintain circadian rhythms by repressing LUX/PRR5/TOC1, consequently reducing *Arabidopsis* freezing tolerance (Wang et al., 2017; Mikkelsen and Thomashow, 2009). CBF1 inhibits its transcription by directly binding to the *LUX* promoter, while in the tea tree, CsCBF1-mediated *CsLUX* activation creates a regulatory node connecting CBF and JA pathways. CsLUX both responds to CsCBF1 and directly modulates JA biosynthesis/signaling by binding *CsLOX2* (JA synthesis enzyme) and *CsJAZ1* promoters, forming a dynamic cold-response network (Wang et al., 2024b).

In addition, cold regulation of alternative splicing of CCA1 and LHY precursor mRNA. Under cold conditions, the splicing form of CCA1 α increases, while the splicing form of CCA1 β decreases. Overexpression of *CBFs* in CCA1 α materials increases the expression level and enhances plant frost tolerance. CCA1 β may inhibit its regulation of *CBFs* by interacting with CCA1 α, negatively regulating plant frost tolerance (Park et al., 2012). A recent study has shown that CCA1 and LHY respond to temperature drops and participate in activating the transcription of flowering regulatory factor VIN3 to cope with long-term cold stress. A vernalization reaction element composed of G-box and EE motifs was discovered in the *VIN3* promoter, which can be recognized by transcription factors CCA1 and LHY, leading to the accumulation of VIN3 during cold stress conditions (Kyung et al., 2022). The results indicate that plants may have integrated their circadian rhythms and flowering components to effectively cope with cold stress. These studies indicate that circadian rhythms are closely related to cold stress. However, further research is warranted on how plants precisely regulate their circadian rhythms under cold stress, and whether there are specific time periods that make plants more sensitive to the circadian rhythm’s response to cold stress.

### 5.4 Hormonal networks coordinate cold stress response

**Figure 6:**
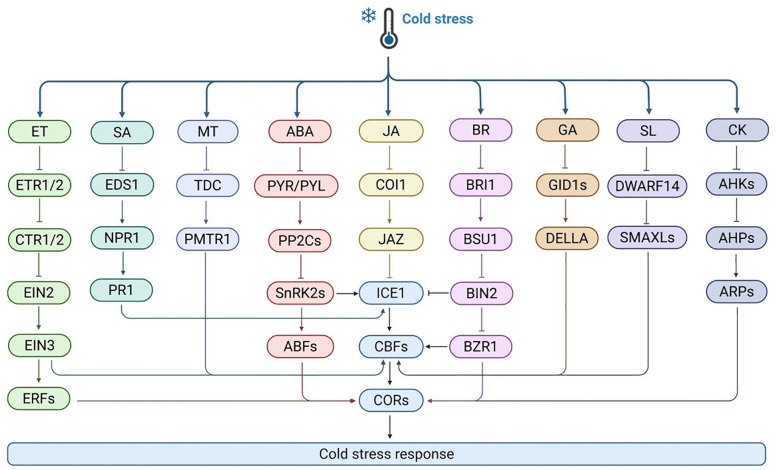
The mechanism of plant respond to cold stress through various hormone signaling pathways. Cold stress activates multiple hormone pathways, including ethylene (ET), salicylic acid (SA), methionine (MT), abscisic acid (ABA), jasmonic acid (JA), brassinosteroid (BR), gibberellin (GA), lactone (SL), and cytokinin (CK). Each pathway perceives signals through its specific receptors (such as ETR1/2, EDS1, PMTR1, PYR/PYL, COI1, BRI1, GID1s, DWARF14, AHKs) and transmits them through intermediate components (such as CTR1/2, NPR1, PP2Cs, SnRK2s, JAZ, BSU1, DELLA, SMAXLs, AHPs), ultimately converging to transcription factors (such as ICE1, CBFs) to activate *CORs* expression and affect plant cold tolerance.

Under cold stress, plant hormones interact to form a complex signaling pathway network that jointly regulates cold response signals. Among these hormones, the CBF gene acts as a vital regulator in hormone crosstalk during the cold stress response. Its expression is regulated by several major plant hormones, including abscisic acid (ABA), brassinosteroid (BR), ethylene (ETH), gibberellin (GA), jasmonic acid (JA). Under cold conditions, the hormonal balance in plants undergoes significant changes (**Figure 6**). The levels of inhibitory hormones, such as ABA, increase, while the levels of growth-promoting hormones, such as IAA and GA, decrease. Plants adapt to cold stress by regulating the proportions of these hormones.

ABA is an endogenous hormone in plants that is widely involved in plant growth, development, and stress response. After cold treatment, the application of exogenous ABA can usually enhance the cold tolerance. the ABA synthesis-deficient mutant *aba3* in *Arabidopsis* exhibited reduced cold tolerance under cold stress, but this deficiency could be compensated for by exogenous ABA treatment (Xiong et al., 2001). The ABA signaling pathway comprises four core components: PYR/PYL/RCAR receptor proteins, PP2C phosphatases, SnRK2 kinases, and ABF/AREB transcription factors. Upon binding to ABA, PYR/PYL/RCAR proteins inhibit the activity of PP2C phosphatases, thereby activating the SnRK2 kinase cascade. SnRK2 kinases, such as OST1, can directly promote the expression of *ICE1*, facilitate the transcription of *CBF* genes, and ultimately enhance plant cold tolerance (Ding et al., 2015). In addition, ABA regulates cold stress response genes through the TOC1-MYB44 module in the CBF-independent pathway, balancing energy allocation and stress adaptation (Du et al., 2024). ABA is also widely involved in other plant responses to cold stress. For example, in rice, ABA induces *OsDREB1B* to enhance cold tolerance by regulating proline synthesis. Overexpression of ABA receptor *PYL10* increases the cold tolerance (Verma et al., 2019). These studies indicate that ABA signaling acts as a crucial regulator in response to cold stress.

BR is a plant specific steroid hormone. After cold treatment, exogenous BR application enhances the expression of *CBF* genes in *Arabidopsis*, indicating that BRs improve frost resistance. BRs bind to the receptor kinase BRI1 and initiate intracellular phosphorylation cascades. In this process, the negative regulator BIN2 undergoes dephosphorylation, activating the transcription factors BES1 and BZR1. Activated BES1/BZR1 regulate many BR response genes. BRI1, after activation, detaches from the BR signal suppressor BKI1 and transmits signals to BSK1 via phosphorylation. BSK1 activates the phosphatase BSU1, which phosphorylates BIN2, inactivating it. This enhances the activity and stability of BZR1 and BES1, enabling them to directly regulate downstream BR gene transcription. Studies show that BR signal-defective mutants are highly freezing-sensitive, with or without cold acclimation. BIN2, a key negative BR signaling factor, undergoes autophosphorylation at low temperatures, inhibiting its binding to the *CBF* promoter and negatively regulating cold stress (Ye et al., 2019). BIN2 overexpression reduces frost resistance, while the *bin2-3bil1-bil2* triple mutant increases it. Unlike BIN2, BES1 and BZR1 positively regulate cold responses (Kim et al., 2024). BZR1, a key BR signaling transcription factor, regulates *CBF1/2* expression by binding to the E-box site in the promoter, actively regulating cold tolerance (Li et al., 2017b). BIN2 can phosphorylate ICE1, enhancing its interaction with HOS1 and destabilizing ICE1. Similar mechanisms exist in other plants. For example, in rice, OsGSK2, the BIN2 homolog, inhibits OsbHLH002 (an ICE1 homolog) transcriptional activity via phosphorylation, reducing *OsCBF3* expression. However, it remains unclear how cold stress directly activate BIN2. CESTA and BZR1, regulated by BR, are BIN2 targets that bind to the *CBF* promoter to regulate constitutive *CBF* expression. OsBRI1, a key BR signaling receptor, causes cold sensitivity when dysfunctional.

Cytokinin is an important hormone in plants, whose transduction operates through a two-component system involving receptor histidine kinase (HK), histidine phosphotransferase (HP), and response regulator (RR). Cold stress can promote an increase in cytokinin levels in *Arabidopsis* roots, thereby activating response RR, which can induce the expression of *SHY2* gene, thereby inhibiting the biosynthesis of auxin and the transport of auxin through PIN1, PIN3, and PIN7. This series of changes will lead to a decrease in auxin levels, causing abnormal root growth. In *Arabidopsis*, ARR1/12 is a key response regulator in the cytokinin signaling pathway. Research has found that ARR1/12 is involved in cold stress mediated root growth inhibition. Specifically, the root length and number of meristematic cells of *arr1–3* and *arr12–1* mutant seedlings are sensitive to cold stress (Zhu et al., 2015). In addition, the decrease in PIN1/3 transcript levels and auxin levels in the roots of *arr1–3 arr12–1* mutant was significantly less. In addition, phosphorylated ARR1 can interact with HPY2, enhance acetylation modification of histone H3, and promote transcriptional activation of ARR1. In maize, under cold stress, the accumulation of ZmRR1 protein significantly increases and induces the expression of *ZmDREB1s* and *ZmCesA* genes, thereby enhancing the cold tolerance of maize (Zeng et al., 2021). Similarly, in rice, OsRR6 can actively respond to cold stress. In plants overexpressing *OsRR6*, the expression of *DREB1A/CBF3*, *COR15A*, *KIN1*, and *RD29A* was significantly upregulated, indicating that OsRR6 plays as a vital regulator in cold response (Bhaskar et al., 2021).

Ethylene signaling receptors include ETR1, ETR2, ERS1, ERS2, and EIN4. In *Arabidopsis*, ethylene typically exhibits negative regulatory effects under cold stress. When ethylene is present, receptors bind to ethylene to inhibit CTR1 kinase activity, activate downstream signal transduction, lead to the accumulation of EIN3 transcription factors, activate *ERF* expression, regulate downstream gene transcription, and enable plants to respond to ethylene signals. In *Arabidopsis*, overexpressed plants of ethylene signaling core factor EIN3 exhibit a phenotype of reduced frost resistance. Further research has shown that EIN3 can bind to the *CBF* promoter and inhibit its expression. In addition, the E3 ligase EBF1/EBF2 in the ethylene signaling pathway negatively regulates plant frost resistance by degrading its target protein EIN3 transcription factor. In rice, EIN family member OsEIN2 is a negative regulator of cold stress. Overexpressed plants of *OsEIN2* exhibit severe stress symptoms at low temperatures, with excessive accumulation of ROS, while *ein2* mutant plants show enhanced cold tolerance. Further research has shown that OsEIL1 and OsEIL2 can form heterodimers and synergistically inhibit the expression of *OsICE1* by binding to their promoters. OsEIN2 and OsEIL1/2 activated by OsICE1 downregulated the expression of *OsICE1* target genes, ROS related, and photosynthesis related genes (Zhai et al., 2024). In addition, overexpression of ethylene related transcription factor VaERF057 can also enhance the cold tolerance. This indicates that the role of ethylene in cold tolerance varies among different plants.

GA is a plant hormone whose signal transduction is centrally regulated by the GRAS protein DELLAs, and participates in response through the GA-GID1-DELLA module during cold stress. In *Arabidopsis*, overexpression of *CBF1* enhances the accumulation of DELLAs by regulating the transcription of RGL3 (Lantzouni et al., 2020). The absence of GA is consistent with the improvement of frost resistance. DELLAs knockout lines *gai-t6* and *rga-24* are sensitive to freezing. Further research has shown that the growth retardation under cold stress is achieved by CBF regulating DELLAs proteins located in the nucleus. The promoting effect of CBF on growth depends on the degradation of DELLAs stimulated by GA. DELLAs protein is a repressor of the GA signaling pathway. GA induces the degradation of DELLAs repressor protein, thereby controlling many key developmental processes and responses to stress such as cold. DELLAs proteins modulate plant cold stress responses through interactions with multiple transcription factors. The interaction between DELLAs and PIF4 can regulate *CBF* transcription. In tomato, SlPIF4 not only actively regulates cold tolerance by directly activating the SlDELLA gene *S1GAI4*, but also activates the gene expression of *SlCBF1* by directly binding to the *SlCBF1* promoter. The crosstalk between PIF4 and DELLAs can regulate *CBFs* transcription and hormone homeostasis in tomato cold response. SlPIF4 not only directly binds to the promoter of *SlCBF1* gene and activates its expression, but also regulates the biosynthesis and signal transduction of plant hormones including abscisic acid, jasmonic acid, and gibberellin to cope with cold stress. In addition, S1PIF4 directly activates the SlDELLA gene S1GAI4 under cold stress, while S1GAI4 positively regulates cold tolerance (Wang et al., 2020).

Melatonin is a widely present active molecule in plants, which helps maintain redox homeostasis by inducing the expression of antioxidant enzyme genes. Exogenous application of melatonin has been shown to enhance the activity of various key antioxidant enzymes. In *Arabidopsis*, MT can upregulate the expression of *CBFs*, *COR15a* and ROS related antioxidant genes, thereby actively regulating plant cold tolerance (Tang et al., 2022). After the melatonin receptor gene CAND2/PMTR1 binds to melanin on the plant cell membrane, it triggers a series of signaling pathways, such as the cAMP pathway and Ca^2+^ signal, to regulate the cold tolerance (Wang et al., 2022; Aghdam and Arnao, 2024). These findings indicate that melatonin can affect plant physiological processes through receptor-mediated signaling pathways. However, research on the molecular mechanisms of melatonin related genes in response to cold stress is limited. Future research on the molecular mechanisms of melatonin in response to cold stress will help us better understand the regulatory mechanisms of melatonin in response to cold stress.

JA is a lipid-derived plant hormone whose levels rise under cold stress. Applying exogenous JA can boost cold tolerance in *Arabidopsis* by increasing *CBF* expression, while impaired JA biosynthesis can make plants more susceptible to freezing. These changes in cold tolerance are mediated by JASMONATE ZIM-DOMAIN (JAZ) proteins, specifically JAZ1 and JAZ4, which act as inhibitors of JA signaling, interacting with ICE1/2 to suppress the transcriptional activity of *ICE1* and the expression of the *CBF1–3* genes (Hu et al., 2013). Additionally, JA-mediated cold-induced growth inhibition is achieved by stabilizing DELLAs proteins, which interact with transcription factors of growth regulatory factors (GRFs) to inhibit their activity. The apple orthologs MdJAZ1/2 exhibit analogous functions by disrupting the MdABI4-MdICE1 interaction. Notably, MdABI4 acts as a positive regulator of MdICE1’s transactivation capacity, with MdJAZ1/2-mediated inhibition consequently attenuating cold tolerance (An et al., 2022).

The SA signaling pathway is essential for cold stress adaptation in plants, where receptor complexes NPR-TGA constitute the central regulatory node. In *Arabidopsis*, SA signaling core receptor NPR1 can sensitively sense cold signals, leading to conformational changes, transitioning from an oligomeric state to a monomeric form and transferring to the nucleus. NPR1 precisely interacts with the transcription factor HSFA1, which can activate *COR* genes expression, significantly enhancing the cold adaptation ability and enabling them to survive and grow under cold conditions (Olate et al., 2018). At the same time, NPR1 can bind to the core transcription factor ICE1 and key transcription factor TGA3 involved in the cold signaling cascade to form a functional protein complex. This complex can directly bind to a specific region of the *PR1* promoter downstream of SA, thereby efficiently activating the transcription of the *PR1* gene. Research has shown that the binding of NPR1 and TGA3 can significantly enhance the transcriptional activation of PR1 by ICE1. This means that under cold conditions, plants can strengthen their immune response in this way, effectively resisting pathogen invasion, which is of great significance for the survival of plants in harsh environments. Similar functional mechanisms have also been found in citrus. CtrNPR3 can interact with CtrTGA2, inhibiting its function and weakening its activation of target genes, thereby exerting a negative regulatory effect on the cold resistance. Additionally, CtrTGA2 can specifically bind to the *CtrP5CS1* promoter and activate the expression. The increased expression of the *CtrP5CS1* gene promotes the synthesis and accumulation of proline, which can help to improve their cold tolerance. CtrTGA2 directly regulates the expression of the gene *CtrICS1* involved in SA biosynthesis, thereby constructing a positive feedback loop (Xiao et al., 2024). In this circuit, the accumulation of SA can further enhance the transcriptional activation of CtrP5CS1 mediated by CtrTGA2, forming a self-enhancing regulatory mechanism and further improving the cold resistance of plants. Interestingly, external application of SA can partially alleviate this inhibitory effect, reactivate the function of CtrTGA2, and enhance the cold resistance of plants. This discovery provides important theoretical basis for regulating plant cold tolerance through exogenous SA.

SL is a sesquiterpene plant hormone that is recognized by D14 (α/β hydrolase) receptors (Umehara et al., 2008). Upon recognition, the D14-SCF-MAX2 complex is formed, targeting the degradation of D53/SMXLs (Waters et al., 2017). The degradation of SMXLs releases the inhibition of downstream *CBFs*, thereby enhancing plant frost resistance. In *Arabidopsis*, cold stress induce the expression of *MAXs* genes and increase endogenous SL levels. SL inhibits the expression of the WRKY41 gene and promotes the interaction between MAX2 and WRKY41. This interaction mediates the degradation of WRKY41 through the 26S proteasome pathway, alleviating the inhibition of WRKY41 on its target gene *CBFs.* As a result, the expression of *CBFs* and their downstream genes enhances the cold tolerance (Wang et al., 2023b).

Of course, there are numerous interactions between hormones and other regulatory factors that collectively respond to cold stress. BIN2 can form a complex with JA signal inhibitor JAZ1 to jointly inhibit ICE1 activity. Exogenous JA treatment can alleviate the cold sensitive phenotype of BIN2 overexpressing strains. Tomato SlWRKY50 promotes cold tolerance by positively regulating the jasmonic acid biosynthesis pathway, and its expression is directly activated by the JA signaling core transcription factor SlMYC2 (Wang et al., 2024a); And OsWRKY53 specifically inhibits the cold tolerance of anthers during the booting stage by antagonizing gibberellin (GA) metabolism. Multiple hormone signaling mediators can specifically attach to the *CBF* promoter region or interact with important regulatory factors of *CBF* expression, thereby promoting plant development during cold acclimation (Tang et al., 2022). However, the complex molecular mechanisms of hormone mediated signaling and its regulation of CBF in cold stress response still need to be elucidated.

## 6 Post-translational regulation in response to cold stress

In addition to transcriptional and post-transcriptional regulation, an increasing body of evidence highlights the significance of post-translational modifications in the cold response, which primarily encompass protein phosphorylation, ubiquitination, and acetylation (Kidokoro et al., 2022).

**Figure 7:**
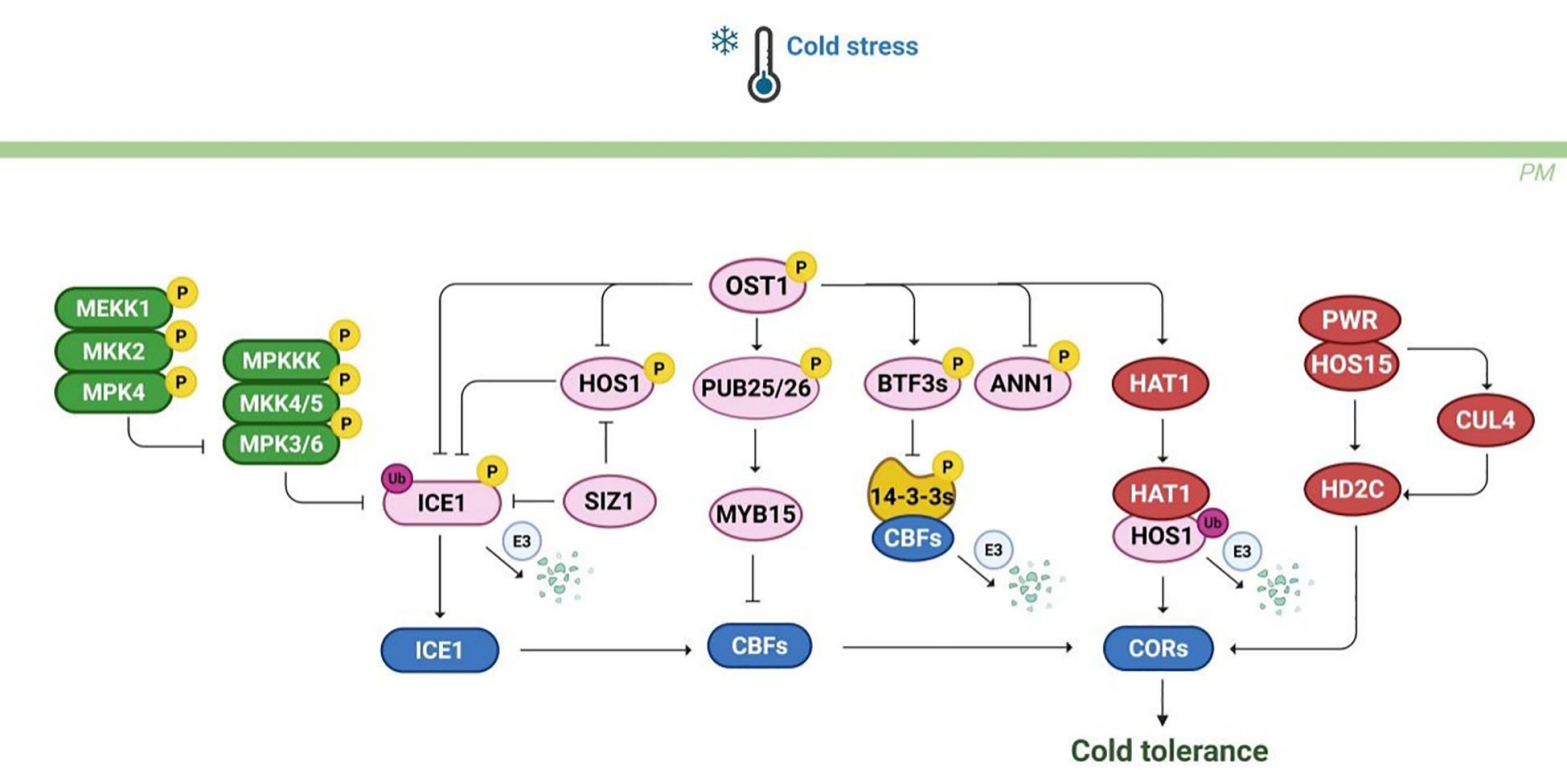
The post-translational modification regulatory network under cold stress. Cold stress activates the MEKK1-MKK2-MPKK4/5-MPK3/6 signaling cascade, phosphorylates ICE1 and promotes its ubiquitination degradation, negatively regulating plant cold tolerance. OST1 responds to cold stress by phosphorylating various regulatory factors such as PUB25/26, BTF3s, ANN1, HAT1. in addition, Regulatory factors such as PWR, HOS15, CUL4, and HD2C regulate *CBF* and *COR* genes through ubiquitination or acetylation modification. In summary, the integration of multiple post-translational modification pathways such as phosphorylation, ubiquitination, and acetylation in plants affects the cold tolerance.

Phosphorylation represents a ubiquitous post-translational modification mechanism essential for signal transduction amplification. OST1, a member of the SNF1-related protein kinase family, is activated under cold stress and regulated by the protein phosphatase CLADE E GROWTH-REGULATING 2 (EGR2). OST1 interacts with the protein phosphatase PP2CG1, thereby inhibiting its activity and negatively regulating cold tolerance (Lv et al., 2021). Moreover, OST1 phosphorylates ICE1 to enhance its stability, thus positively responding to cold stress. OST1 also mediates the interaction between HOS1 and ICE1, which effectively prevents the degradation of ICE1 by HOS1 (Ding et al., 2015). Additionally, OST1 phosphorylates the U-box E3 ligases PUB25 and PUB26, thereby enhancing their E3 activity. This leads to the polyubiquitination and degradation of MYB15, which in turn boosts *CBF* expression under cold stress (Wang et al., 2019b). OST1 can also interact with a newly formed peptide-associated complex (NAC) protein BTF3s and phosphorylate it, promoting its interaction with CBF proteins to enhance frost resistance (Ding et al., 2018). Interestingly, OST1 can interact with AtANN1 and phosphorylate it, thereby augmenting its Ca^2+^ transport activity and enhancing Ca^2+^ signaling (Liu et al., 2021a). These findings demonstrate that OST1 responds to cold stress via multiple pathways. Recent research indicates that OST1 mediates acetylation in response to cold stress (**Figure 7**). Specifically, OST1 phosphorylates the histone acetyltransferase HAT1, promoting its interaction with HOS1. This interaction induces the ubiquitination and degradation of HAT1, thereby alleviating the inhibition of CBF genes by HAT1 and activating the cold stress response (Kang et al., 2025).

The MAPK cascade also transduces cold stress signals in plants via sequential phosphorylation events. This cascade comprises three core kinase families: MAP3K, MAP2K, and MAPK. In *Arabidopsis*, two well-characterized MAPK cascades are the MEKK1-MKK1/2-MPK4 and MKK4/5-MPK3/6 pathways. Under cold stress conditions, MEKK1 kinase activity is upregulated, which leads to the phosphorylation of MKK2 and subsequently activates MPK4 and MPK6. Notably, the MEKK1-MKK2-MPK4 pathway positively regulates cold tolerance by inhibiting MPK3/MPK6 activity. In contrast, MPK3/6 negatively regulate cold responses by phosphorylating ICE1, thereby reducing its stability (Li et al., 2017a). Additionally, MPK6 phosphorylates MYB15, which negatively regulates *CBF3* gene expression (Kim et al., 2017). MAPK signaling is also implicated in cold responses across various plant species. In tomato, SlMPK1 and SlMPK2 enhance cold tolerance by phosphorylating the transcription factor SlBBX17. In rice, OsMAPK3 improves cold resistance by phosphorylating OsICE1, which inhibits its ubiquitination and activates the expression of OsTPP1 (Zhang et al., 2017). Furthermore, the kinase OsCTK1 phosphorylates OsMPK1, which in turn dephosphorylates MAPK3/6, thereby positively regulating cold tolerance (Wu et al., 2024). Recent studies have identified the phosphatase OsPP2C27 negatively regulates cold tolerance by directly dephosphorylating OsMAPK3 and OsbHLH002 (Xia et al., 2021). In summary, the MAPK signaling cascade is critically involved in mediating plant cold response. However, further investigation is required to fully understand species-specific variations in MAPK-mediated cold signaling. Gaining deeper insights into these regulatory pathways will be essential for developing crop varieties with improved cold tolerance.

Ubiquitination is another important post-translational modification involved in cold stress. In *Arabidopsis*, E3 ubiquitin ligase HOS1 degrades ICE1 through ubiquitination, thereby reducing its stability at low temperatures (Dong et al., 2006). SIZ1 inhibits the 26S proteasome degradation of ICE1 protein by SUMOylation, reducing HOS1 mediated polyubiquitination to improve stability (Miura et al., 2007). Similarly, in apple, the interaction between MdSIZ1 and MdMYB2 significantly enhances plant cold tolerance. Further research suggests that inducing MdMYB2 at low temperatures can activate *MdSIZ1* expression, thereby promoting the synthesis of anthocyanin biosynthesis (Jiang et al., 2022). However, the substrates regulated by ubiquitinases under cold stress are currently limited, and further exploration is needed for the identification of targets through ubiquitination modification. In addition, E3 ubiquitinase is a key factor in ubiquitination modification, but the regulatory mechanism of E3 ubiquitinase under cold stress is not fully understood.

Substantial evidence indicates that histone acetylation modifications directly correlate with cold tolerance capacity across plant species. This reversible modification system operates via the counterbalancing activities of histone acetyltransferases (HATs) and histone deacetylases (HDACs), which precisely tune acetylation homeostasis to regulate transcriptional reprogramming during cold stress. These enzymes modulate gene expression by interacting with transcription factors involved in cold signaling. Generally, histone acetylation promotes gene transcription, whereas deacetylation leads to transcriptional repression. In *Arabidopsis*, the histone deacetylase HD2C is closely linked to the CBF-dependent pathway. Under normal conditions, HD2C collaborates with HOS15 to suppress the expression of *COR* genes by deacetylating their promoters. However, under cold stress, the PWR-HOS15 complex facilitates the degradation of HD2C, enabling the recruitment of CBF to the *COR* gene promoters and promoting their expression (Lim et al., 2020). Additionally, HOS15 recruits the CUL4 ubiquitin ligase to degrade HD2C, thereby increasing H3 acetylation at *COR* promoters and enhancing cold tolerance. Similarly, in rice, OsHDA716 interacts with OsbZIP46, which leads to the deacetylation of the OsbZIP46 DNA-binding domain, reducing its binding affinity and transcriptional activity. Consequently, the stability of OsbZIP46 is compromised, resulting in decreased cold tolerance (Sun et al., 2024).

Beyond the CBF-dependent pathway, histone acetylation is also involved in CBF-independent cold stress responses. For instance, in *Arabidopsis*, the interaction between RHOMBOID-like protease 11 (RBL11) and fatty acid export protein 1 (FAX1) promotes the ubiquitination and degradation of FAX1, which is vital for cold adaptation (John et al., 2024). Moreover, the transcription factor ARABIDOPSIS RESPONSE REGULATOR 1 (ARR1) is regulated through phosphorylation and SUMOylation, which influence its activity and promote H3 acetylation, thereby modulating cold-responsive gene expression. In apple, the histone deacetylase MdHDA6 negatively regulates cold tolerance by deacetylating and repressing the expression of MdTCP15, a negative regulator of cold stress. MdTCP15 directly binds to the promoter of *MdCOR47* and reduce cold tolerance (Guo et al., 2023). Collectively, these findings highlight the significant role of histone acetylation in plant responses to cold stress. However, the number of identified genes regulated by histone acetylation under cold conditions remains limited, and the underlying regulatory mechanisms require further investigation.

## 7 Balancing cold stress and multiple traits

When plants cope with cold stress, they need to finely balance and regulate cold tolerance with other traits. This trade-off mainly relies on two types of genes: “traditional” trade-off genes and “ideal” trade-off genes. The former is prone to negative effects when balancing traits, while the latter can achieve trait balance while maintaining the original state of the plant (Yang et al., 2025). Exploring “ideal” genes is significant for cultivating new varieties with cold tolerance.

CBF is a typical “traditional” trade-off gene. In *Arabidopsis*, overexpression of *CBF* constitutively activates cold stress response, which increases plant resistance to cold stress but also leads to growth inhibition, accumulation of DELLA protein, and inhibition of stem elongation (Achard et al., 2008). Similarly, high expression levels of *CBF1/3* genes can lead to similar problems. Overexpression of *CBF3* gene in cassava improves the cold resistance of transgenic cassava, but also introduces adverse effects such as growth retardation, leaf curling, shortened root length, and reduced yield (An et al., 2016).

In crops, mining and utilizing “ideal” trade-off genes could enhance cold tolerance in plants without compromising growth or yield, providing new strategies for cultivating crop varieties with traits such as cold tolerance and high yield. For example, the maize heat shock transcription factor HSF21 is considered as an “ideal” trade-off genes. HSF21 enhances the chilling tolerance by maintaining lipid metabolism homeostasis and modulating natural genetic variation under cold stress. Overexpression of HSF21 not only enhances maize cold tolerance, but also significantly increases maize field yield. Further analysis of high-throughput corn lipidomics revealed that HSF21 is a key gene regulating lipid metabolism homeostasis under cold stress, controlling the metabolic balance of unsaturated lipids under cold stress (Gao et al., 2024).

## 8 Discussion

Over recent decades, substantial advancements have been achieved in elucidating plant responses to cold stress. Early investigations primarily examined low-temperature-induced alterations in physiological and biochemical characteristics, such as membrane lipid phase changes. Subsequent progress in molecular techniques redirected attention to the isolation and functional characterization of cold-responsive genes. Critical regulators like CBF/DREB transcription factors were identified, offering mechanistic insights into cold adaptation pathways. Nevertheless, plant cold stress adaptation constitutes a multifaceted biological phenomenon, with existing knowledge representing merely an initial exploration of this intricate system.

From the perspective of research history, early studies often focused on a single gene, but plant cold response clearly involves the interaction of multiple genes and signaling pathways. Future research requires the use of systems biology methods to integrate multiple omics data and construct a complete cold responsive molecular network. Therefore, future research should focus on the following aspects: firstly, the initial perception mechanism of cold signals. The cell membrane is the main sensing site, but there is still no answer on how cold stress activates cold signal receptors and whether receptors depend on ROS or NO signaling. The second is the modification mechanism of CBF protein. The newly discovered modification pathway, such as histone acetylation, is involved in CBF regulation, but its specific mechanism is still unclear. The third is how plants balance key regulatory factors under cold stress. Light, flowering, circadian rhythm, hormones, and other factors are widely involved in the cold response, but their equilibrium mechanisms are still unclear.

At present, most existing research is based on the model plant *Arabidopsis*. With the intensification of global climate change, the frequency and intensity of extreme cold weather are fluctuating, posing new threats to crop production. Studying how crops perceive and respond to cold stress, and how to regulate the balance between growth and resistance, is crucial for cultivating cold resistant varieties and ensuring food security. At the same time, in actual agricultural production, there are many types of crops, and the cold tolerance of different crops varies greatly. It is necessary to strengthen the exploration of cold responsive genes specific to different crops and their application in breeding. This is expected to cultivate crop varieties with strong cold tolerance and excellent performance in key indicators such as yield, thereby promoting higher levels of agricultural production, meeting the growing demand for food in the future, and addressing complex and changing environmental challenges.

In addition, the long-term adaptation mechanism of plants to cold stress also deserves further research. Most studies have focused on the short-term response of plants to cold stress, lacking systematic research on how plants maintain growth and development, complete their life cycle and other issues in long-term cold condition. Meanwhile, the interaction between cold stress and other environmental stresses is also a direction for future research. In natural environments, plants often face a combination of various stresses, such as cold-drought, cold-salt stress, etc. Studying the response mechanisms of plants under these complex stresses is of great significance for a comprehensive understanding of plant environmental adaptability.

In summary, although the research on plants’ responses to cold stress has achieved fruitful results, it still faces many challenges. Through interdisciplinary collaboration and advanced research techniques and methods, we are expected to gradually uncover the mysteries of plant response to cold stress, providing a solid theoretical foundation and technical support for cultivating cold resistant crops and addressing climate change.
